# The global battle for sexual rights

**DOI:** 10.7448/IAS.17.1.19243

**Published:** 2014-05-17

**Authors:** Dennis Altman, Chris Beyrer

**Affiliations:** 1 Office of the Vice Chancellor, La Trobe University, Melbourne, Australia;; 2 Department of Epidemiology, Johns Hopkins Bloomberg School of Public Health, Baltimore, MD, USA

Increasingly, people across the world are being persecuted on account of their sexuality or, in some cases, their presumed sexuality or gender identity. In Cameroon, four women have recently been arrested and imprisoned on vague charges of lesbianism; in Nigeria, men have been whipped for homosexuality, while crowds called for their execution. Unverified numbers of men have been executed for homosexuality in Iran; and draconian anti-homosexual laws have been introduced in Russia and Uganda, leading to people fleeing the country for fear of their lives. The Supreme Court of India has recently upheld colonial era laws criminalizing homosexual behaviour; in Malaysia, the government consistently attacks both homosexuals and transgender people. Similar laws are being mooted in parliaments in Kenya, Ethiopia, Ghana and other nations.

At the same time, there is a remarkable shift in attitude in most of what used to be called the western world, symbolized by the growing number of jurisdictions that recognize same sex marriage (including several conservative American states), and strong official statements against homophobia in a number of Latin American countries. Few issues, indeed, so polarize the international arena as does homosexuality.

In 2011, the United Nations Human Rights Council (UNHRC) resolved that human rights should apply without distinction on the basis of sexual orientation or gender identity. In response, Pakistan's envoy claimed that “licentious behaviour promoted under the concept of ‘sexual orientation’ is against the fundamental teachings of various religions including Islam,” and led a coordinated walkout from the discussion. On several occasions, Secretary General Ban Ki Moon has spoken strongly in favour of protecting the human rights of all people irrespective of their sexuality or gender identity.^1^

Over the past few years, there has been a marked increase in anti-homosexual legislation in some African and Arab states, while Vladimir Putin has made opposition to “advocacy” of homosexuality a major issue within Russia, fuelling vigilante activities. There is on-going persecution of homosexuals in Jamaica, and Brunei has recently mooted introducing sharia law which would involve stoning to death those involved in same-sex conduct. What Tom Boellstorff, originally writing on developments in Indonesia, termed “political homophobia,” is spreading, and it is almost always linked to the rhetoric of opposing degenerate western influence (1). This is particularly ironic as it means anti-colonial governments across much of the former British Empire are defending laws that were introduced by the colonial power (which long ago have been repealed), while much of the fervour for attacking homosexuality comes from American evangelists. Indeed, American pastor, Scott Lively, who is currently being sued in Massachusetts for his involvement in the Ugandan law, has worked closely with anti-homosexual groups in both Africa and Russia.

Those who work in HIV care have long been aware of the difficulties for health promotion these sorts of laws and persecution create, and there is considerable evidence that, even in states which are not actively persecuting homosexuals, far fewer resources are devoted to reaching people vulnerable to infection through homosexual contact than the epidemiology demands (2). But, in the face of a determination to portray homosexuality as a mark of unwanted western degeneracy, rational arguments about public health carry little weight. And pointing to the high rates of HIV transmission through male to male behaviour can easily be turned into more arguments to stigmatize and punish.

Equally, indignation and even threats to cut development assistance from donors too often backfire. When David Cameron, the British Prime Minister, mooted withdrawing aid from African countries because of their anti-homosexual policies, gay groups in those countries rightly pointed to the fact this was likely to increase persecution and the perception that they were, indeed, a foreign import. Although it has been heartening to see such strong support for sexual and gender rights from the Obama Administration, statements of support for sexual rights can easily be twisted as yet more evidence that the west is promoting alien lifestyles, and can be used to entrench the very governments which are being criticized.

Much as we are tempted to express outrage at the abuses being perpetrated in the name of protecting morality, it is always incumbent upon those of us who live in comparatively safe and supportive environments to ask what the impact of our actions will be upon those who are being targeted. The recent decision of the US Administration to cut aid to the Inter-Religious Council of Uganda, which has supported anti-homosexual laws, does retain grants for treatments, and several European governments have also cut aid strategically, redirecting the funds to NGOs working in the country. By themselves such actions will do nothing to weaken the authoritarian control of President Museveni, although support for local community based groups is crucial.

Because unscrupulous leaders will appeal to religion, tradition and culture to denigrate people who do not fit the heteronormative order, they need be countered by recognizing the strength of traditional assumptions about gender and sexuality, and the genuine fears of many people when these norms come under attack. Western governments and movements that are leading calls for boycotts and cuts to aid should at least reflect on their own history, and the reality that during the George W. Bush Administration, the United States aligned with Iran and the Vatican to block any mention of homosexuality at the special session of the General Assembly on AIDS.

The ultimate arguments against persecution—which can take the form of murder, torture and “corrective rape” of women presumed to be lesbian—require a careful and culturally aware engagement with notions of normality and bodily autonomy. It is important to counter the myths that homosexuality is a foreign import, and to stress that it is part of the natural variety of human sexual expression found across history and geography. It is equally important to link homosexuality to a broader conception of human rights, which is based upon the dignity of every individual and her or his right to control of her or his body as long as no harm is done to others.

At this point in history, sexuality has become a polarizing issue in the international arena, and both sides are tempted to play to their domestic audiences rather than consider the human costs. There is an urgent need for strategic reflection on how best to promote greater acceptance of sexual rights in a world in which too often sexuality has become a touchstone for fear, prejudice and hatred. Too often silence in the face of increasing persecution is the result of indifference, not a strategic decision about how best to address injustice.

## Note

1 There are considerable number of speeches by the Secretary General and the High Commissioner for Human Rights Navi Pillay available on line. See also on-going posts by Charles Radcliffe from the global issues section at the UN human rights office in New York on the *Huffington Post* online.
